# Assessing the genetic relationship between phimosis and 26 urogenital diseases: a Mendelian randomization study

**DOI:** 10.3389/fendo.2024.1308270

**Published:** 2024-06-10

**Authors:** Wei Li, Ying Yu, Hu Li, Xingliang Yang, Tao Li

**Affiliations:** ^1^ Department of Urology, The Affiliated Hospital of Guizhou Medical University, Guiyang, China; ^2^ Emergency Department, Affiliated Hospital of Binzhou Medical College, Binzhou, China; ^3^ Department of Urology, Urologic Surgery Center, Xinqiao Hospital, Third Military Medical University (Army Medical University), Chongqing, China; ^4^ State Key Laboratory of Trauma and Chemical Poisoning, Third Military Medical University (Army Medical University), Chongqing, China

**Keywords:** phimosis, urogenital system, Mendelian randomization, urinary tract infection, malignant tumors of the urogenital system

## Abstract

**Purpose:**

This study aims to investigate the impacts of phimosis on the health of the genitourinary system through Mendelian random analysis.

**Material and method:**

A dual-sample Mendelian randomization (MR) analysis was conducted using the publicly available genome-wide association study (GWAS) data. The inverse variance weighted based on the random effects model (Re-IVW) method was used as the main statistical analysis. Complementary methods, including weighted median, MR-Egger regression, and MR pleiotropy residual sum and outlier (MR-PRESSO), were applied to detect or correct the impact of horizontal pleiotropy.

**Result:**

Re-IVW showed a genetic predictive causal relationship of phimosis on glomerulonephritis (odds ratio [OR]: 1.37 [1.13–1.65], *p* = 0.00149) and IgA glomerulonephritis (OR: 1.57 [1.18–2.09), *p* = 0.00187). Suggestive evidence indicated that phimosis was associated with chronic nephritis syndrome (OR: 1.23 (1.00–1.51), p = 0.0481], acute nephritis syndrome (OR: 1.50 [1.13–2.01], *p* = 0.0058), and impotence (OR: 1.39 [1.11–1.73], *p* = 0.0035). Kidney and ureteral stone (OR: 1.14 [1.04–1.26], *p* = 0.0069), urethral strictures (OR: 1.26 [1.07–1.48], *p* = 0.0050), benign prostatic hyperplasia (OR: 1.07 [1.01–1.13], *p* = 0.0242), and decreased testicular function (OR: 0.72 [0.56–0.94], *p* = 0.0141) have genetically predictive causal relationships.

**Conclusion:**

In summary, we employed a series of reliable analytical methods to investigate the association between phimosis and 26 urogenital diseases. We have reported several strong associations, but more research is needed to evaluate whether this discovery is replicated in other environments and to gain a better understanding of potential mechanisms.

## Introduction

Phimosis refers to a narrow opening of the foreskin that cannot be retracted to expose the glans penis (1, 2). This can have complex interactions with the genitourinary system, potentially increasing the risk of complications such as wrapping balanitis, urinary retention, urinary tract infection, erectile dysfunction, male infertility, and urological tumors (3, 4). For instance, numerous observational studies revealed that patients who have not undergone circumcision have a cumulatively higher risk of developing penile cancer than the general population (4–6). However, previous epidemiological studies claimed that the USA (which has a high rate of circumcision) showed similar penile cancer risk compared with Denmark (which has a low rate of circumcision) (7). In addition, research regarding the association between phimosis and other urogenital diseases is relatively limited. Due to the varied mixed factors, contradictory conclusions have been reported (8–11), and the causal relationship between phimosis and the risk of urogenital diseases is still unclear. Nonetheless, considering the prevalence of phimosis worldwide, it is meaningful to conduct large-scale and effective randomized controlled trials (RCT) to clarify the relationship between phimosis and urogenital health. However, the costs, logistical issues, and some interventions that are not approved or suitable for RCT evaluation have made the RCTs difficult to conduct, making clarifying the role of phimosis in male reproductive health more difficult.

Recently, Mendelian randomization (MR) analysis has become a popular and effective method for causal reasoning. It uses genetic variation (single nucleotide polymorphism [SNP]) as the instrumental variable (IV) to explore the causal relationship between results and exposures (12, 13), which effectively avoids bias in traditional epidemiological research, providing a valuable alternative to randomized clinical trials. We plan to investigate the causal relationship between phimosis and 26 urogenital diseases (testicular hypofunction, testicular dysfunction, male infertility, impotence, abnormal spermatozoa, kidney stones, calculus of the lower urinary tract, retention of urine, urethral stricture, hydronephrosis, glomerulonephritis, lgA glomerulonephritis, acute nephritic syndrome, chronic nephritic syndrome, nephrotic syndrome, acute renal failure, chronic kidney disease, cystitis, prostatitis, urethritis, orchitis and epididymitis, malignant neoplasm of the kidney, malignant neoplasm of the prostate, malignant neoplasm of the testis, malignant neoplasm of the bladder, and prostatic hyperplasia) through MR analysis to comprehensively explore the effects of phimosis on urogenital health.

## Method

### Research design

The design of this study referred to the Report List of Mendelian Randomization-Enhanced Epidemiological Observational Studies (STROBE-MR) (13). We conducted a dual-sample MR study using data from 27 publicly aggregated genome-wide association study (GWAS) statistics (one exposure and 26 outcomes), with these cohorts limited to subjects of European descent to reduce population stratification bias. All the data used in this work came from studies with subject consent and ethical recognition. Therefore, our study does not require ethical approval from the institutional review committee.

### Data source

FinnGen research is a unique study that combines genomic information with digital healthcare data from participants aged 18 and above residing in Finland (14). Among the 27 GWAS datasets involved in this study, abnormal spermatozoa were obtained from FinnGen (seventh edition), while the rest were obtained from FinnGen (ninth edition), and the detailed description of these GWAS datasets involved in this study is included in the supplementary documents (**Supplementary Table S1**).

### Selection of IV

For the selection of instrumental variables, we follow the following criteria: (1) independent SNPs (*r* ^2^ = 0.001, KB = 10,000) with locus-wide significance (*p* < 1*e*−06); (2) nonrare SNPs (minor allele frequency [MAF] ≥ 0.05); (3) unrelated SNPs unrelated to potential confounders (diabetes, smoking, and body mass index) by checking each of the SNPs in the PhenoScanner database (http://www.phenoscanner.medschl.cam.ac.uk/). After obtaining a reliable SNP through the appeal criteria, we use F statistics to estimate the strength of each genetic instrument and delete SNPs with lower genetic strength (*F* < 10) (15). The formula is *R* ^2^ × (*N* − 2)/(1 − *R* ^2^), where *R* ^2^ is the cumulative explained variance of selected SNPs in exposure that used (2 × EAF × (1 − EAF) × beta^2^)/[(2 × EAF × (1 − EAF) × beta^2^) + (2 × EAF × (1 − EAF) × *N* × SE(beta)^2^)], where *N* is the sample size of research, EAF is the effect allele frequency, beta is the estimated genetic effect, and SE (beta) is the standard error of the beta. The last 24 SNPs were retained and used for subsequent analysis (**Supplementary Table S2**).

### MR analysis

All analyses were performed in R software (version 4.2.3) using the R package “TwoSampleMR” (version 0.5.6). The Re-IVW (as the main analysis) is used to summarize the Wald ratio for each SNP, allowing for heterogeneity between SNPs and returning unbiased estimates of causal relationships when all IVs are valid and the level of pleiotropy is balanced (16, 17). The weighted median (18) and MR-Egger (19) methods, which make diverse assumptions about horizontal pleiotropy, were performed as complementary methods to test the robustness of the main analysis. Cochran’s *Q* statistic (20) was applied to evaluate heterogeneity. The MR-Egger intercept method (21) and the leave-one-out method (22) were used to evaluate horizontal pleiotropy. MR pleiotropy residual sum and outlier (MR-PRESSO (23) can identify outliers in horizontal pleiotropy and correct possible distortions caused by outliers. *p* < 0.05 was considered nominally significant, whereas the level for statistical significance corrected for multiple testing (1 exposure × 26 outcomes = 26 tests) was set at *p* = 0.05/26 = 1.92*E*−03. *p* < 0.05 is considered significant in heterogeneity and pleiotropy analyses.

## Result

### Phimosis and male reproductive health

**Figure 1:**
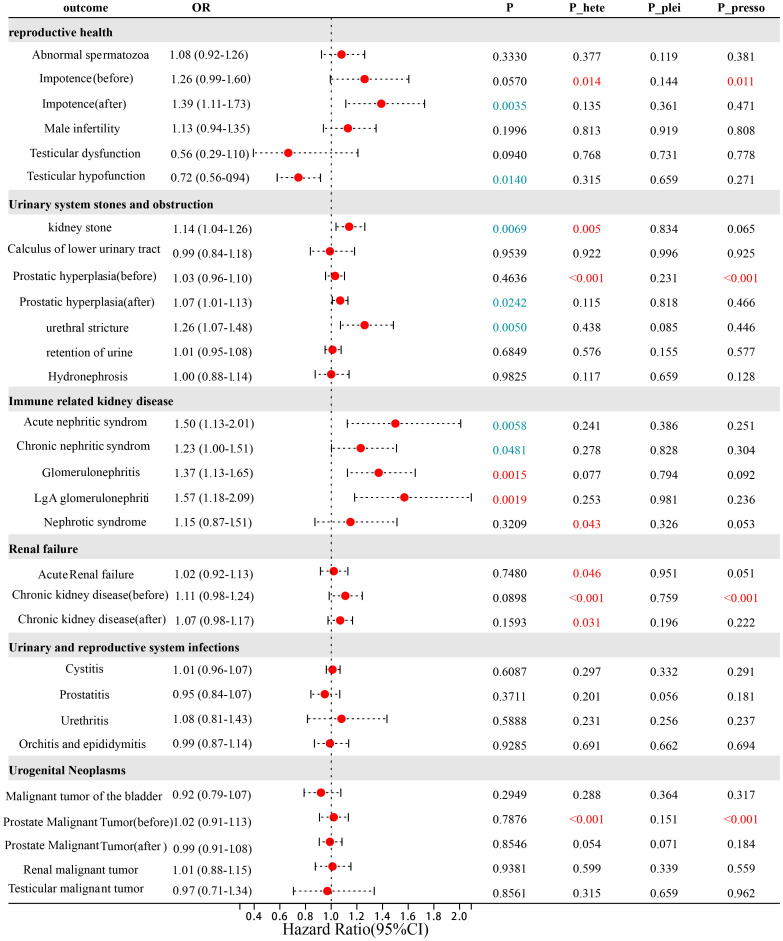
The genetically predictive causal relationship between phimosis and 26 urogenital diseases by Re-IVW. P_plei is the result of the Egger intercept test; P_hete is the result of Cochrane’s *Q* test; P_presso is the result of MR_presso.

We did not observe a causal relationship between phimosis and male reproductive diseases (**Figure 1**). However, suggestive evidence indicates an association between phimosis and testicular hypofunction (odds ratio [OR]: 0.72 [0.56–0.94], *p* = 0.0141). Cochran’s *Q* statistic found that only impotence had heterogeneity. We observed the heterogeneity disappeared after excluding abnormal SNPs (rs376877), and a suggestive relationship between phimosis and impotence (1.39 [1.11–1.73], *p* = 0.0035). Meanwhile, the MR-Egger intercept and leave-on-out analyses did not find potential level pleiotropy, confirming the reliability of our results.

### Phimosis urolithiasis and urinary obstruction

Here, we did not observe a causal relationship between phimosis and these diseases (**Figure 1**). However, indicative evidence presented phimosis had causal relationships with urethral stricture (OR: 1.26 [1.07–1.48], *p* = 0.0050) as well as kidney stone (OR: 1.14 [1.04–1.26], *p* = 0.00669). Although the subsequent Cochran’s *Q* statistic revealed heterogeneity in benign prostatic hyperplasia (BPH) and kidney stones, it did not impair the reliability of this study. Afterwards, MR-PRESSO revealed two abnormal SNPs (rs3130593 and rs3873444) for BPH. After excluding these SNPs, the heterogeneity disappeared, and a suggestive causal relationship was found between phimosis and BPH (OR: 1.07 [1.01–1.13], *p* = 0.0242) (**Figure 1**). Finally, the effect of horizontal pleiotropy was also not found.

### The causal relationship between phimosis and immune-related kidney disease

We noticed that phimosis promotes the occurrence of glomerulonephritis (1.37 [1.13–1.65], *p* = 0.00149)) and IgA glomerulonephritis (1.57 [1.18–2.09], *p* = 0.00187) (**Figure 1**), and suggestive evidence also suggests that phimosis promotes the occurrence of acute/chronic nephritis syndrome, with OR values of 1.50 (1.13–2.01, *p* = 0.0058) and 1.23 (1.00–1.51, *p* = 0.0481), respectively (**Figure 1**). Cochran’s *Q* statistic found heterogeneity in the nephrotic syndrome. However, MR-PRESSO did not find any abnormal SNPs. Meanwhile, the MR-Egger intercept method and the leave-one-out method confirmed the reliability of our results.

### The causal relationship between phimosis and renal failure

We did not observe that phimosis has an impact on renal function (**Figure 1**). Although heterogeneity testing revealed significant heterogeneity in both acute renal failure and chronic kidney disease, the results after removing abnormal SNPs (rs2071479) in chronic kidney disease are also consistent with those before, indicating the reliability of our results. At the same time, the MR-Egger intercept method and the missed one method have also confirmed that our results are not affected by horizontal pleiotropy.

### The causal relationship between phimosis and urinary and reproductive system infections

We did not find any evidence to suggest (**Figure 1**) that phimosis is associated with urinary and reproductive system infections. Subsequently, Cochran’s *Q* statistic, MR-PRESSO, MR-Egger intercept method, and leave-one-out method confirmed the reliability of our results.

### The causal relationship between phimosis and malignant tumors of the urinary and reproductive systems

We did not find a causal relationship between phimosis and malignant tumors of the urinary and reproductive systems (**Figure 1**). Although the Cochran’s *Q* statistic found significant heterogeneity in prostate malignant tumors, the weighted median and the result after excluding abnormal SNPs (rs3130593, rs9267529, rs376877, and rs4985030) were also consistent with previous results. Meanwhile, the MR-Egger intercept and leave-one-out analysis also confirm the reliability of our results.

## Discussion

A dual-sample MR analysis was used to evaluate the causal relationship between phimosis and 26 urogenital diseases in this study. We found that phimosis was positively correlated with glomerulonephritis and IgA glomerulonephritis. Meanwhile, suggestive evidence showed that phimosis was positively correlated with chronic nephritis syndrome, acute nephritis syndrome, impotence, kidney and ureteral stones, urethral stricture, prostate hyperplasia, and urinary retention; meanwhile, it was negatively correlated with testicular hypofunction.

Phimosis is considered a risk factor for several urogenital diseases (3, 4). However, previous observational studies always drew conflicting conclusions due to various confounding factors (8–12). We observed a clear causal relationship between phimosis and immune-related kidney disease, but relevant research was lacking and the specific mechanism was unknown (24, 25). As the direct virulence stimulation of bacteria and viruses, as well as chronic inflammatory and oxidative stress damage caused by infection, have been proven to increase the incidence of immune-related kidney disease, we speculated the phimosis-caused infection might be the underlying mechanism (26–29). However, more effective clinical and mechanistic studies are warranted to clarify this issue.

Impaired male reproductive ability was another concern for patients with phimosis; impaired sexual function can lead to a series of physical and mental illnesses (30), which impels more individuals to receive circumcision. Although some studies have shown that phimosis impairs penile erection (31), others have reported controversial results. On the contrary, some patients complained of abnormal sexual sensations or requiring more effort to achieve orgasm after circumcision, which was related to partial nerve loss (10, 11). In this study, we found a causal relationship between phimosis and impotence through MR analysis and observed suggestive evidence to confirm that phimosis increased impotence risk. Moreover, we also present that phimosis revealed protective effects on testicular function, which have never been reported, and the specific mechanism was even more unclear.

Most observational studies have claimed that phimosis increases infection risks and leads to chronic inflammatory stimulation, which might contribute to urogenital diseases (9, 24, 25, 32). However, we did not find a direct causal relationship between phimosis and four urogenital infections (prostatitis, testicular and epididymitis, urethritis, and cystitis). This suggested that the phimosis-related infection was not related to genetic factors; it might come from indirect factors like patients who did not receive circumcision had lower education or income level, paid less attention to genitourinary health, and possessed more unhealthy behaviors such as masturbation (33, 34). Our research suggested that phimosis might play a more essential role in the genitourinary system but the current research was limited, and the specific mechanism was unclear. However, considering the prevalence of phimosis, we should not overlook the impact of phimosis on the genitourinary system.

We explored the relationship between phimosis and 26 urogenital diseases through MR analysis, which is currently the most comprehensive and first relevant study. Secondly, the design of MR analysis is not easily affected by confounding factors. We eliminated the potential impact of pleiotropy on the results by using multiple MR methods, the PhenoScanner database, and removing SNPs related to known risk factors. Therefore, our results are unlikely to be interfered with by horizontal pleiotropy. In addition, the genetic variation between phimosis and 26 urogenital diseases is derived from summary-level data from GWAS, which has a large sample size and can reduce the impact of confounding factors. Finally, this study revealed a possible causal relationship between previously unreported phimosis and other urogenital diseases, which may inspire future research.

However, there are several limitations in this study. First, all GWAS data come from the population of European ancestry; whether this result can be extended to the whole population should be cautioned. Second, although our study has a large sample size, the several genetic tools used for exposure and outcomes are to varying degrees affected by low statistical power and incomplete phenotype definitions, which may lead to ineffective findings in most of the associations explored and cannot distinguish causal relationships between different periods. Therefore, a larger GWAS for phimosis with more precise phenotypic definitions would be beneficial. Thirdly, we cannot rule out the possibility that our research results may be affected by weak instrument bias, which depends on a relatively relaxed threshold of *p* = 1 × 10^−6^ chosen genetic instruments, although the F statistical data did not indicate that our tools were weak. Last but not least, the potential mechanism mediating the causal relationship between phimosis and 26 urogenital diseases has not been studied, and further functional research is needed.

## Conclusion

In summary, we employed a series of reliable analytical methods to investigate the association between phimosis and 26 urogenital diseases. We have reported several strong associations, but more research is needed to evaluate whether this discovery is replicated in other environments and to gain a better understanding of potential mechanisms.

## Data availability statement

The original contributions presented in the study are included in the article/**Supplementary Material**. Further inquiries can be directed to the corresponding author.

## Author contributions

WL: Conceptualization, Data curation, Writing – original draft, Writing – review & editing. YY: Writing – review & editing. HL: Writing – review & editing. XY: Writing – review & editing. TL: Conceptualization, Data curation, Methodology, Writing – original draft, Writing – review & editing.
