# Supplementary material for: Assessing the genetic relationship between phimosis and 26 urogenital diseases: a Mendelian randomization study
Source: Front Endocrinol (Lausanne). 2024 Jun 10;15:1308270. doi: 10.3389/fendo.2024.1308270 (PMC11194306; doi:10.3389/fendo.2024.1308270)
Supplement: Supplementary file 2 [file Table_2.docx]

#* --- Load the required packages -------------------------------------

library (TwoSampleMR)

library(data.table)

library(tidyverse)

library(readxl)

library(writexl)

library (MRInstruments)

library(plyr)

library(dplyr)

#* ---Obtaining instrumental variables-------------------------------------

GWAS<-fread("Phimosis.gz")

exp_a<-subset(GWAS , pval<1e-06)

exp_data <- clump_data(exp_data , clump_kb = 10000,clump_r2 = 0.001)

write.csv(exp_data, ‘IV related to phimosis.csv’)

#*-note: Calculate the F value of the saved file in Excel and remove unreliable SNPs according to the filtering criteria in the text

#* ---Set working directory-------------------------------------

`%+%` <- function(x,y) paste0(x,y)

dir.create(getwd()%+%"/data")

path_data <- getwd()%+%"/data"

dir.create(getwd()%+%"/result")

path_res <- getwd()%+%"/result"

##-note: The data folder contains a summary of GWAS data for 26 urogenital diseases

#* --- #* --- Preparation before running loop code-------------------------------------

FileNames<-list.files(paste0(/data),pattern=".gz")

mr_presso<-list()

plot1<-list()

plot2<-list()

IV<- read.csv(‘IV related to phimosis.csv’)

IV$ phenotype <-‘phimosis’

exp_data<-format_data(IV,

type="exposure",

phenotype_col = " phenotype",

snp_col = "SNP",

beta_col = "beta",

se_col = "se",

pval_col = "p",

effect_allele_col = " effect_allele ",

other_allele_col = " other_allele ")

#* --- #* --- Run MR analysis and save the results -------------------------------------

for(i in c(1:length(FileNames))){

GWAS_1 <- fread(paste0(path_data,"/",FileNames[i]))

exp<-strsplit(FileNames[i], '.gz')[[1]][1]

comsnp<-intersect(exp_data$SNP,GWAS_1$rsids)

GWAS_2<-subset(GWAS_1,rsids%in%comsnp)

GWAS_2<-as.data.frame(GWAS_2)

out_data <- format_data(GWAS_2,

type="outcome",

snp_col = "rsids",

beta_col = "beta",

se_col = "sebeta",

eaf_col = "af_alt",

pval_col = "pval",

effect_allele_col = "alt",

other_allele_col = "ref",)

dat <- TwoSampleMR::harmonise_data(

exposure_dat = exp_data,

outcome_dat = out_data)

#### Direct removal of palindromes

dat <-subset(dat,mr_keep==TRUE)

res=TwoSampleMR::mr(dat)

print(paste0(exp,"_SNP数_",res$nsnp[1]))

results <- TwoSampleMR::generate_odds_ratios(res)

results$estimate <- paste0(

format(round(results$or, 2), nsmall = 2), " (",

format(round(results$or_lci95, 2), nsmall = 2), "-",

format(round(results$or_uci95, 2), nsmall = 2), ")")

resdata <- dat

names(resdata)

Assumption13 <- subset(resdata,mr_keep==TRUE,

select = c("SNP","pval.exposure",

"pval.outcome", # "F_statistic",

"mr_keep"))

res_hete <- TwoSampleMR::mr_heterogeneity(dat)

res_plei <- TwoSampleMR::mr_pleiotropy_test(dat)

res_leaveone <- mr_leaveoneout(dat)

openxlsx::write.xlsx(x = list(

"main"=results,

"Assumption13"=Assumption13,

"pleiotropy"=res_plei,

"heterogeneity"=res_hete,

"leaveone"=res_leaveone),

overwrite = TRUE,

paste0("result/",exp,"-res.xlsx"))

p1<-mr_scatter_plot(mr_results = mr(dat,method_list = c("mr_ivw","mr_egger_regression","mr_weighted_median")),dat)

plot1[[i]]<-p1

p2<-mr_leaveoneout_plot(leaveoneout_results = mr_leaveoneout(dat))

plot2[[i]]<-p2

res_presso <- TwoSampleMR::run_mr_presso(dat,

NbDistribution = 1000)

mr_presso[[i]]<-res_presso

}

names(mr_presso)<-FileNames

names(plot1)<-FileNames

names(plot2)<-FileNames

saveRDS(mr_presso,'mr_presso.RDS')

saveRDS(plot1,'plot1.RDS')

saveRDS(plot2,'plot2.RDS')

##-note: According to the results of MR_presso, the results after excluding abnormal SNPs need to be analyzed repeatedly
